# Advancements and Applications of Liquid Biopsies in Oncology: A Narrative Review

**DOI:** 10.7759/cureus.42731

**Published:** 2023-07-31

**Authors:** Jawad Noor, Ahtshamullah Chaudhry, Riwad Noor, Saima Batool

**Affiliations:** 1 Internal Medicine, St. Dominic Hospital, Jackson, USA; 2 Medicine/Public Health, Nishtar Hospital, Multan, PAK; 3 Pathology, Nishtar Medical University, Multan, PAK

**Keywords:** application, advancements, precision medicine oncology, oncology, liquid biopsy

## Abstract

According to the World Health Organization (WHO), nearly 10 million people died from cancer worldwide in 2020, making it the leading cause of mortality. Liquid biopsies, which provide non-invasive and real-time monitoring of tumor dynamics, have evolved into innovative diagnostic techniques in the field of oncology. Liquid biopsies offer important insights into tumor heterogeneity, treatment response, minimum residual disease identification, and personalized treatment of cancer through the analysis of circulating tumor DNA (ctDNA), circulating tumor cells (CTCs), extracellular vesicles, and microRNAs. They offer several advantages over traditional tissue biopsies, such as being less invasive, more convenient, more representative of tumor heterogeneity and dynamics, and more informative for guiding personalized treatment decisions. Liquid biopsies are being utilized increasingly in clinical oncology, particularly for patients with metastatic disease who require ongoing monitoring and treatment modification. In this narrative review article, we review the latest developments of liquid biopsy technologies, their applications and limitations, and their potential to transform diagnosis, prognosis, and management of cancer patients.

## Introduction and background

Cancer is a diverse and complicated illness caused by genetic and epigenetic changes in normal cells. These changes provide cancer cells with aberrant growth, survival, invasion, and metastatic capacities, making them difficult to identify and treat. Traditionally, cancer diagnosis and characterization have relied on tissue biopsies, which include the removal of a sample of tumor tissue and subsequent processing in the laboratory [1]. Additionally, tissue biopsies could miss the tumor's spatial and temporal heterogeneity, which can change and evolve over time in response to medical interventions or external stimuli. Therefore, there is an unmet need for alternative methods to diagnose and monitor cancer that are less invasive, more convenient, more representative, and more informative. Liquid biopsies are emerging as a promising technique to address this need. Several Food and Drug Administration (FDA)-approved diagnostic tests are available to detect cancer and determine its prognosis, offering valuable insights. The provision of information and recommendation of optimal treatment to patients can aid in mitigating the likelihood of disease development through lifestyle modifications and medical interventions [2].

The field of liquid biopsy has witnessed remarkable advancements, fueled by rapid technological developments in molecular biology, genomics, and next-generation sequencing [3]. As liquid biopsies advance, they have the potential to change cancer diagnosis and management by giving healthcare professionals the ability to gather real-time information on tumor dynamics and enabling individualized treatment plans.

## Review

How do liquid biopsies have advantages over traditional tissue biopsies?

When diagnosing cancer, tissue biopsies are considered the "gold standard," as they are currently the most reliable method. In tissues where sample extraction is challenging, liquid biopsy has become a viable alternative to aid in the diagnosis of primary tumors or the assessment of the stage of metastatic illness. Moreover, it can mitigate the complications associated with invasive tissue biopsy, including bleeding, infections, and pain [4]. Unlike tissue biopsies, which need invasive procedures to collect samples from particular tumor locations, liquid biopsies use readily available body fluids. This accessibility enables repeated sampling over time, facilitating monitoring and treatment adjustment.

Liquid biopsy is a novel approach for detecting the presence of tumor recurrence and real-time monitoring of tumor dynamics and treatment response by identifying particular markers in body fluid samples, in contrast to traditional biopsies that involve direct examination of tumor tissue [3]. It is important to note that even if a tumor exists, a single liquid biopsy sample may not disclose it, so a confirmatory test with a tissue biopsy may be needed [5].

Analytes used in liquid biopsy

Liquid biopsy uses various types of analytes. These analytes include circulating tumor cells (CTCs), circulating tumor DNA (ctDNA), and cell-free DNA (cfDNA) in blood or other fluid samples [3]. It offers an alternative to conventional tissue biopsies, facilitating real-time monitoring of disease progression and treatment. These analytes provide useful information regarding tumor characteristics, genetic alterations, treatment response, and progression [6]. These advantages are achieved without the requirement of invasive procedures. Table 1 shows various types of analytes and their applications used in liquid biopsy.

**Table 1:** Various types of analytes and their applications used in liquid biopsy. CTCs: circulating tumor cells; cfDNA: cell-free DNA; EVs: extracellular vesicles; ctDNA: circulating tumor DNA; miRNAs: microRNAs.

Analytes	Description	Applications
CTCs [6]	Cancer cells detached from the primary tumor and enter the bloodstream for analysis	- Provides information about the tumor's genetic profile - Assesses metastatic potential - Evaluates drug resistance
cfDNA [7]	Small DNA fragments released into the bloodstream by normal and cancerous cells	- Provides information about the tumor's genetic profile - Assesses metastatic potential - Evaluates drug resistance
EVs [8]	Small membrane-bound vesicles released by cells, including cancer cells	- Offers insights into the tumor's molecular characteristics - Reveals intercellular communication
ctDNA [9]	Subset of cfDNA originating from tumor cells, carrying tumor-specific genetic alterations	- Detects and monitors cancer - Identifies mutations and copy number variations - Aids in treatment selection
miRNAs [10]	Small non-coding RNA molecules involved in gene regulation, with altered expression patterns associated with various diseases, including cancer	- Identifies disease-associated miRNA expression patterns - May serve as potential biomarkers for cancer and other diseases

Significance of CTCs in tumor biology

Tumor biology focuses on understanding the biological processes involved in cancer development and progression. It investigates various aspects of tumor cells, interactions with the surrounding environment, and tumor growth and metastasis mechanisms. Metastasis is a crucial step in cancer progression. It is a complex process involving multiple steps that can differ across different types of cancer. Understanding the molecular and cellular events of metastasis helps to develop effective treatments. Studying CTCs provides valuable insights into the metastatic cascade and potential target interventions. It includes information from initial tumor cell extravasation to the circulation and formation of clinically detectable metastases [11].

Novel high-resolution techniques, such as single-cell sequencing, next-generation sequencing (NGS), and microfluidic platforms, analyze the genomes and transcriptomes of CTCs, providing detailed information about the genetic makeup and gene expression patterns and uncovering important molecular features associated with metastasis and drug resistance. Capturing viable CTCs facilitates the development of culturing technologies to study the fundamental characteristics of CTCs, such as invasiveness, their kinetics, and how they respond to selection barriers like therapeutic interventions [12].

Exploring the Role of CTCs in the Disease Progression

CTCs exhibit invasiveness as they detach from the primary tumor, infiltrate the surrounding tissues, and gain access to the bloodstream, thereby increasing the potential for metastasis. CTCs are identified in the peripheral blood of most cancer patients, including people with localized disease and those at risk of recurrence after treatment. CTCs are sparse, with only one CTC in a million blood cells in patients [12].

In local invasion, cancer cells break surrounding structural barriers and tissues such as the extracellular matrix (ECM), basement membranes, or neighboring cells. This process involves the activation of specific signaling pathways, such as transforming growth factor-beta (TGF-β), epidermal growth factor receptor (EGFR), Notch, and nuclear factor-kappa B (NF-κB), and the secretion of enzymes, such as matrix metalloproteinases (MMPs), cathepsins, serine proteases (including urokinase-type plasminogen activator (uPA) and tissue-type plasminogen activator (tPA)), hyaluronidase, and A disintegrin and metalloproteinases (ADAMs), that degrade the ECM, allowing cancer cells to invade adjacent tissues [13]. Specific signaling pathways and enzymes involved in local invasion vary and depend on the tumor's cancer type and individual characteristics.

Intravasation refers to the process by which CTCs enter the blood vessels. The invading cancer cells penetrate blood or lymphatic vessels entering the circulatory system. Intravasation can occur through various mechanisms, including active migration or passive entry during the remodeling of tumor-associated blood vessels [14]. For survival, CTCs evade immune detection by downregulating major histocompatibility complex (MHC) molecules and inhibiting immune cell recognition while utilizing immunosuppressive cells like regulatory T cells (Tregs) and myeloid-derived suppressor cells (MDSCs) to inhibit immune responses, with antigens such as programmed death ligand-1 (PD-L1) and cytotoxic T-lymphocyte-associated antigen-4 (CTLA-4) inhibiting T-cell function [15]. CTCs release immunosuppressive factors like TGF-β and interleukin-10 (IL-10) to suppress dendritic cell functioning and reduce the immunity of effector CD4+ and CD8+ T cells [16].

CTCs reduce physical stress in the circulatory system through shape, flexibility, and adhesion changes, enhancing resistance to mechanical stresses while also potentially secreting proteins and activating signaling pathways that promote cell survival, including heat shock proteins Hsp90 and 70 [17]. Some CTCs may form clusters, where multiple cancer cells adhere to each other or to other blood components, providing protection and enhancing their survival capabilities. CTCs stick through E-cadherin and integrins and form clusters with platelets that offer security, improving CTC survival. E-selectin facilitates CTC attachment to endothelial cells and platelet-derived growth factor (PDGF), promoting CTC survival [18].

CTCs exit the bloodstream through the extravasation process and enter new tissues at distant sites to metastasis. The extravasation process is similar to the invasion step, as cancer cells must penetrate the target organ's vessel walls, invading the surrounding tissue. CTCs establish themselves in the new tissue microenvironment during extravasation and form metastatic lesions. This step requires cancer cells to adapt to the specific conditions of the target organ, including interactions with resident cells, angiogenesis induction, and evasion of immune responses. The successful colonization of CTCs leads to the growth of secondary tumors and the progression of metastatic disease [19].

Traditionally, the detection of CTCs involved invasive procedures such as bone marrow aspiration. However, the development of liquid biopsy techniques has provided a non-invasive alternative for CTC detection. Various techniques, including immunomagnetic separation, microfluidic technologies, and size-based filtration systems, capture and characterize CTCs based on physical or biological properties. Physical methods include size-based filtration, density gradient centrifugation, and separation based on electrical properties. Biological methods include affinity-binding properties of CTCs using specific biomarkers to selectively capture them [20].

Epithelial cell adhesion molecule (EpCAM) is a frequently used biomarker for CTCs articulated on epithelial cells. EpCAM-based methods may have limitations for aggressive cancers, as sometimes they do not exhibit EpCAM expression. To overcome the challenge of detecting EpCAM-negative CTCs, methods such as depleting CD45-positive leukocytes or utilizing multi-marker antibody combinations are employed [21].

Role of ctDNA as a surrogate marker for tumor genetic alterations

cfDNA is a mixture of regular and tumor-derived DNA called ctDNA. It was first discovered in 1948, and in 1977, tumor patients were found to have significantly higher levels of cfDNA, which was later identified in 1989 to originate from tumor cells [22]. Limited detection methods hindered research progress with cfDNA, but in 1994, specific gene mutations were identified, providing a basis for tumor diagnosis, and in 1999, abnormal DNA methylation was detected in non-small cell lung cancer (NSCLC) patients using methylation-specific PCR [22]. In 2005, ctDNA mutations were assessed in a clinical setting for the first time. The clinical validation of ctDNA to detect EGFR mutations was approved in 2014 [23].

A liquid biopsy can identify ctDNA, which is derived from tumor cells. It is a non-invasive method that analyzes components in bodily fluids like blood or urine. Techniques such as NGS, polymerase chain reaction (PCR), and droplet digital PCR (ddPCR) are used to detect and analyze ctDNA. This information helps diagnose cancer, select treatments, and monitor tumor dynamics [24]. ctDNA is a short DNA fragment released by tumor cells carrying genetic alterations specific to the tumor. ctDNA serves as a surrogate marker for tumor genetic alterations in liquid biopsy. Surrogate biomarker genes have expedited gene transfer evaluation, improving effectiveness, location, and duration, enabling gene therapies [25].

cfDNA is released by both non-cancerous cells and tumor cells. ctDNA is considered as 0.01-5% of cfDNA. Analyzing ctDNA allows for the characterization of biological cancer profiles and the monitoring of cancer progression. ctDNA has a relatively short half-life of approximately two hours [25]. A blood sample is taken, and ctDNA is detected using various methods, such as ultrasensitive targeted PCR.

Targeted PCR-based approaches involve the amplification of a specific target DNA sequence of interest. Examples of such techniques include allele-specific PCR (AS-PCR), digital PCR (dPCR), ddPCR, allele-specific amplification refractory mutation system (ARMS) PCR, and BEAMing (beads, emulsion, amplification, and magnetics) [26].

NGS is a robust and high-throughput technique to sequence DNA rapidly and generate large amounts of data, enabling simultaneous detection of the genome and multiple rare mutations of ctDNA without the need for sequencing the primary tumor such as tagged-amplicon deep sequencing (TAm-Seq), Safe-Sequencing System (Safe-SeqS), and CAncer Personalized Profiling by deep sequencing (CAPP-Seq) [23]. Untargeted techniques, such as whole-genome sequencing (WGS) or whole-exome sequencing (WES) techniques, allow for the detection of novel and clinically significant genomic aberrations without requiring specific information about the primary tumor.

Exosomes

Liquid biopsy identifies exosomes to detect tumors by carrying specific cancer-related proteins or biomarkers. The most common biomarkers are tumor-specific antigens such as HER2/neu for breast cancer, genetic mutations such as EGFR mutation of lung cancer, or cancer-associated proteins like PSA for prostate cancer. Liquid biopsy helps exosomes with isolation and characterization, making the identification of tumors possible [27].

Tumor-educated platelets

Platelets are crucial for tumor recognition through liquid biopsy. Platelets are "scanning soldiers" during immunological and inflammatory responses associated with diseases like tumor progression. The relationship between platelets and tumor growth has been discovered since Trousseau's observations in 1865, who suggested platelets can pick up RNA-containing vesicles secreted by cancer cells [26].

Platelet mRNA profiles are used for cancer diagnostics. These cells participate in systemic and local responses to tumor growth and metastasis. Along with other biomarkers such as CTCs, ctDNA, and exosomes, tumor-educated platelets (TEPs) are an advancement of technology propelling liquid biopsy into a new era, holding promise for cancer diagnosis, screening, and therapy monitoring by liquid biopsy [26].

Liquid biopsy applications

Early Cancer Detection and Identification of Minimal Residual Disease

Minimal residual disease (MRD) is the presence of residual tumor cells or sparse malignant cells that cannot be identified through standard radiological examinations or conventional techniques. Liquid biopsy has the potential to detect MRD. It analyzes tumor-specific alterations in the blood through CTC, ctDNA, or tumor-specific microRNA (miRNA). DNA methylation alterations can also be detected in liquid biopsy. Abnormal methylation patterns in cancer cells have shown promising results as an independent predictive marker for the high risk of relapse in specific types of cancer. However, the specificity of methylation pattern-based MRD detection is insufficient for routine clinical use [28].

High-Risk Population Screening

Liquid biopsy is potentially used for screening high-risk populations for early cancer detection. High-risk populations are more susceptible to cancer development due to previous cancer treatments, family history, genetic mutations, and exposure to carcinogens, which involves analyzing the circulatory metabolites of malignant neoplasm or other body fluids [29]. RNA-Seq analysis of platelet-derived RNA can detect both early- and late-stage NSCLC with an accuracy of around 80%, which involves sequencing the RNA molecules of platelets and analyzing gene expression patterns [28]. It shows promise as a non-invasive diagnostic tool for NSCLC, enabling early detection.

Disease Recurrence Monitoring

Liquid biopsy is also used for monitoring any signs of disease recurrence. It detects the emergence of treatment-resistant clones. By examining ctDNA, CTCs, or other biomarkers in the circulation, liquid biopsies provide a non-invasive and dynamic tool for monitoring disease development [23]. By periodically analyzing these biomarkers, liquid biopsy can offer valuable insights into the efficacy of ongoing treatments by monitoring alterations in the tumor's genetic makeup or the presence of specific mutations linked to resistance to therapy.

Treatment Response Monitoring

ctDNA monitoring has also shown utility in evaluating therapeutic response for MRD and its recurrence after chemotherapy. Somatic mutations, such as BRAF and RAS detection in ctDNA, improve treatment response and survival outcomes. ctDNA levels predict tumor response to immunotherapy and evaluate markers such as microsatellite instability (MSI) and tumor mutational burden (TMB) [29]. The presence of specific exosomal miRNAs, long non-coding RNAs (lncRNAs), and circular RNAs (circRNAs) are biomarkers for diagnosing cancer, predicting prognosis, and assessing treatment response.

Identifying Resistance Mechanisms

Liquid biopsy for identifying resistance mechanisms depends on individualized health and cancer type. Like in NSCLC targeted therapy and immunotherapy from liquid biopsy, specific gene alterations and mutations of EGFR, rearrangements of the anaplastic lymphoma kinase (ALK), and ROS proto-oncogene 1 (ROS1) provide guidance for treatment decisions [30]. Resistance mechanisms to targeted therapies, such as secondary mutations or membrane receptor amplification, are explored. Valuable tools for detecting these resistance mechanisms and guiding further treatment options are emphasized.

In context to immunotherapy, utilizing immune checkpoint inhibitors (ICIs), which may target programmed cell death protein 1 (PD-1) and PD-L1, yields promising results in a subset of patients, with only around 30% achieving durable clinical responses [31]. Liquid biopsy can evaluate patients more likely to respond to ICIs depending on their biomarkers, such as PD-L1 expression and TMB [32].

Sensitivity and specificity of liquid biopsy-based assays

The overall sensitivity of liquid biopsies ranges from 60% to 85%, according to a Cure article published in April 2020. Its specificity and sensitivity vary according to tumor type, patient health, or other clinical factors [33].

Klein et al. researched a blood-based liquid biopsy test using cfDNA sequencing and machine learning, with a sensitivity of 51.5% and specificity of 99.5% across cancer types, but the test had a limited success rate for detecting early-stage cancers (16.8% for stage I) due to lower ctDNA release [34].

PanSeer, developed by Singlera Genomics in the USA, is a blood-based screening test that utilizes ctDNA methylation for early-stage cancer detection, with an overall specificity of 96.1% and a sensitivity of 87.6% for post-diagnosis samples and a sensitivity of 94.9% for pre-diagnosis samples showing promising results for five cancer types replacing conventional biopsies [35]. 

The accuracy of liquid biopsies is not always reliable for cancer diagnosis, as physicians most likely suggest having tissue biopsies after liquid biopsy results come back positive. Liquid biopsy may miss genetic changes at early stages because of low ctDNA concentration [36].

Integration of liquid biopsy into personalized treatment approaches

Liquid biopsy allows a comprehensive genetic profile of the tumor, including information about the tumor's mutations, genetic alterations, and clonal evolution, to identify potential therapeutic targets. It can access tumor heterogeneity by capturing genetic material shed by multiple tumor sites for understanding the different molecular characteristics of various tumor subclones, guiding treatment strategies targeting specific subclones, and monitoring the response of each subclone to therapy.

Liquid biopsy enables real-time monitoring of genetic changes throughout treatment, which is crucial to identify emerging resistance mechanisms, new mutation acquisition, and MRD helping in decision-making and timely adjustments to treatment plans, including the selection of alternative therapies or combination treatment strategies.

Identification of Actionable Mutations and Predictive Biomarkers

Liquid biopsy is a non-invasive method that identifies specific genetic changes in ctDNA, known as actionable mutations. By analyzing ctDNA, liquid biopsy can also detect predictive biomarkers such as high tumor mutational burden (TMB), microsatellite instability (MSI), or specific genetic alterations. These biomarkers indicate the likelihood of a patient responding well to particular treatments, including immunotherapy [37]. This information helps in guiding treatment decisions and selecting therapies with a higher potential for success [37].

Optimizing Regimens and Minimizing Adverse Effects

Dynamic monitoring of treatment response and detecting emerging resistance mechanisms help optimize treatment regimens by adjusting the dosage, selecting, adding, switching therapies, or combining treatments to overcome resistance and maximize effectiveness.

Liquid biopsy has helped minimize adverse effects, avoiding unnecessary treatments for patients. Identifying predictive biomarkers causative of toxicity or lack of response helps professionals make informed decisions about treatment options with a higher likelihood of success while minimizing potential side effects [38].

Limitations of liquid biopsies

Liquid biopsies have shown immense potential in the field of oncology. However, they are not yet considered a standard diagnostic tool. Several issues have to be overcome before they can be widely used. These challenges include uncertainty regarding representative sampling of all genomic clones within a tumor, difficulties in detecting rare components, the need for cost-effective pre-profiling strategies, fragility of some biomarkers, lack of standardized methods, occurrence of false-positive and false-negative results, influence of microenvironmental factors, challenges posed by heterogeneity and count of CTCs [39], and the potential need for an initial histological examination by tissue biopsy. Overcoming these challenges is crucial to ensure the reliability, accuracy, and clinical utility of liquid biopsies.

Liquid biopsy advancements and considerations

Liquid biopsy's future hinges on addressing technical considerations, including standardization, sensitivity, and cost-effectiveness. Standardized protocols and methodologies are crucial for consistent and reliable results. Improving the sensitivity and specificity of the assay is crucial for accurately detecting low levels of biomarkers.

Cost-effectiveness should be enhanced through efficient workflows, robotics, and optimized assay costs. These advancements can make liquid biopsy a valuable tool in personalized medicine for non-invasive disease monitoring, treatment response, and identifying actionable biomarkers [39].

The future of liquid biopsies requires rigorous validation through large-scale clinical trials to enhance clinical utility and efficacy. Studies involving diverse patient populations and comparing them with traditional biopsy methods are necessary to evaluate their sensitivity, specificity, and predictive value. Robust evidence will enable confident integration of liquid biopsy into routine clinical practice, facilitating informed diagnosis, treatment selection, and monitoring decision-making.

Establishing the clinical utility of liquid biopsy in routine oncology practice requires prospective studies to evaluate patient outcomes and cost-effectiveness. Collaborative efforts are needed to establish guidelines for its appropriate use. By demonstrating its benefits, liquid biopsy has the potential to transform cancer management by providing non-invasive, real-time molecular information to guide treatment decisions and improve patient outcomes [40].

The future of liquid biopsies holds potential for multi-analyte profiling and integration with omics technologies. Expanding beyond ctDNA, analysis of CTCs, extracellular vesicles, and tumor-derived proteins can provide a comprehensive tumor profile. Integrating liquid biopsy with transcriptomics, proteomics, and metabolomics enhances understanding of cancer mechanisms and improves personalized treatment strategies [41].

## Conclusions

In conclusion, liquid biopsy is a valuable non-invasive tool for medical diagnostics, mainly when traditional tissue biopsies are impractical or invasive. It analyzes circulating biomarkers in bodily fluids, offering early disease detection and comprehensive assessment of tumor genetic heterogeneity. Although the success rate currently ranges between 60% and 80%, ongoing research aims to enhance sensitivity and accuracy. With advances in genomic sequencing, novel biomarker identification, and integration of artificial intelligence, liquid biopsy has the potential to transform clinical practice, enabling personalized medicine and improving patient care.
